# Prediction of ovarian cancer using artificial intelligence tools

**DOI:** 10.1002/hsr2.2203

**Published:** 2024-06-28

**Authors:** Seyed Mohammad Ayyoubzadeh, Marjan Ahmadi, Alireza Banaye Yazdipour, Fatemeh Ghorbani‐Bidkorpeh, Mahnaz Ahmadi

**Affiliations:** ^1^ Department of Health Information Management, School of Allied Medical Sciences Tehran University of Medical Sciences Tehran Iran; ^2^ Health Information Management Research Center Tehran University of Medical Sciences Tehran Iran; ^3^ Department of Obstetrics and Gynecology Tehran University of Medical Sciences Tehran Iran; ^4^ Students' Scientific Research Center (SSRC) Tehran University of Medical Sciences Tehran Iran; ^5^ Department of Health Information Technology, School of Paramedical and Rehabilitation Sciences Mashhad University of Medical Sciences Mashhad Iran; ^6^ Department of Pharmaceutics and Pharmaceutical Nanotechnology, School of Pharmacy Shahid Beheshti University of Medical Sciences Tehran Iran; ^7^ Medical Nanotechnology and Tissue Engineering Research Center Shahid Beheshti University of Medical Sciences Tehran Iran

**Keywords:** artificial intelligence, machine learning, ovarian cancer

## Abstract

**Purpose:**

Ovarian cancer is a common type of cancer and a leading cause of death in women. Therefore, accurate and fast prediction of ovarian tumors is crucial. One of the appropriate and precise methods for predicting and diagnosing this cancer is to build a model based on artificial intelligence methods. These methods provide a tool for predicting ovarian cancer according to the characteristics and conditions of each person.

**Method:**

In this study, a data set included records related to 171 cases of benign ovarian tumors, and 178 records related to cases of ovarian cancer were analyzed. The data set contains the records of blood test results and tumor markers of the patients. After data preprocessing, including removing outliers and replacing missing values, the weight of the effective factors was determined using information gain indices and the Gini index. In the next step, predictive models were created using random forest (RF), support vector machine (SVM), decision trees (DT), and artificial neural network (ANN) models. The performance of these models was evaluated using the 10‐fold cross‐validation method using the indicators of specificity, sensitivity, accuracy, and the area under the receiver operating characteristic curve. Finally, by comparing the performance of the models, the best predictive model of ovarian cancer was selected.

**Results:**

The most important predictive factors were HE4, CA125, and NEU. The RF model was identified as the best predictive model, with an accuracy of more than 86%. The predictive accuracy of DT, SVM, and ANN models was estimated as 82.91%, 85.25%, and 79.35%, respectively. Various artificial intelligence (AI) tools can be used with high accuracy and sensitivity in predicting ovarian cancer.

**Conclusion:**

Therefore, the use of these tools can help specialists and patients with early, easier, and less expensive diagnosis of ovarian cancer. Future studies can leverage AI to integrate image data with serum biomarkers, thereby facilitating the creation of novel models and advancing the diagnosis and treatment of ovarian cancer.

## 1 INTRODUCTION

Cancer is a malignancy characterized by high aggressiveness, low survival rates, and prolonged and costly treatment procedures. The disease's high recurrence and mortality rates make it imperative to achieve early detection and precise prognostication of cancer, as these measures are crucial for improving the likelihood of patient survival.^1^, ^2^, ^3^ Ovarian cancer ranks among the most common types of cancer impacting women. Every year, over 240,000 new cases of ovarian cancer are identified, and approximately 150,000 women lose their lives to this disease. Ovarian cancer consists of a diverse group of tumors that are categorized based on distinct histopathological and molecular characteristics. Epithelial ovarian cancer (EOC) is the predominant type of ovarian cancer. It can be categorized into four main subtypes based on tumor cell appearance: serous, endometrioid, clear cell, and mucinous. The significant morbidity and mortality associated with ovarian cancer can be attributed to the late detection of the disease and reduced effectiveness of surgical or pharmacological treatments. Ovarian cancer often presents with symptoms that appear late and are not specific, leading to up to 75% of cases being diagnosed at an advanced stage. Unfortunately, only about 20% of those diagnosed at this stage will survive for 5 years after diagnosis.^4^, ^5^

Different screening techniques like pelvic exams, transvaginal ultrasounds, CA125 cancer antigen tests, and magnetic resonance imaging (MRI) imaging are used to identify this disease. However, using any of these methods may not guarantee accurate diagnosis. For instance, pelvic examination and ultrasound have low sensitivity and specificity, while CA125 marker levels may not rise in all patients with ovarian cancer. Furthermore, an expert specialist is required for accurate diagnosis through MRI imaging, which can be challenging. Additionally, there is no proof of cost‐effectiveness associated with any of these diagnostic methods.^6^, ^7^, ^8^ The development of predictive tools has enabled patients and medical practitioners to carry out diagnostic procedures more accurately and quickly while also enabling them to devise treatment plans that are well‐suited to the specific needs of each patient. Artificial intelligence (AI) systems have gained widespread adoption as a result of their numerous benefits and can be employed to surmount the shortcomings of traditional diagnostic techniques. These systems have several advantages, such as their ability to handle large quantities of data, address instances of missing data, and adapt to new data inputs.^9^, ^10^

AI techniques have been increasingly utilized in recent times for precise diagnostic applications across diverse disease categories. In recent years, various AI tools, especially machine learning (ML) and deep learning have become popular for diagnosing and predicting various diseases, especially cancer, due to their advantages. For this reason, many studies have been published in this field.^1^, ^7^ In addition, limited studies have been conducted concerning the prediction of ovarian cancer employing AI (ML) tools. However, due to the restrictions of these studies, the need for newer and more complete studies is felt.^11^, ^12^

Therefore, this study proposes the adoption of artificial intelligence‐based systems as prediction tools for ovarian cancer. In this regard, a set of data will be extracted from a data set including the information of different patients, and AI methodologies will be employed to construct diversified models that can effectively predict ovarian cancer. The best‐performing model will then be identified through subsequent evaluations.

## 2 METHODS

### 2.1 Data set

This study was conducted in 2022–2023. These data are available in a Mendeley Data repository “Using Machine Learning to Predict Ovarian Cancer”^13^ located at https://data.mendeley.com/datasets/th7fztbrv9/11 for any academic, educational, or research purposes. The data set includes the data of 349 patients with 49 characteristics as input (Table 1).

**Table 1:** Data set description.

Feature name	Unit	Type	Range	Missing values
Alpha‐fetoprotein (AFP)	ng/mL	Real	[0.610–508]	24
Anion gap (AG)	mmol/L	Real	[6.200–33.330]	1
Age	year	Integer	[15–83]	0
Albumin (ALB)	g/L	Real	[22–51.500]	10
Alkaline phosphatase (ALP)	u/L	Integer	[26–763]	10
Alanine aminotransferase (ALT)	u/L	Integer	[4–86]	10
Aspartate aminotransferase (AST)	u/L	Integer	[7–78]	10
Basophil cell count (BASO #)	10^9^/L	Real	[0–0.120]	0
Basophil cell ratio (BASO%)	%	Real	[0–1.940]	0
Blood urea nitrogen (BUN)	mmol/L	Real	[1.120–10.190]	0
Calcium (Ca)	mmol/L	Real	[0.920–2.830]	0
Carbohydrate antigen 125 (CA125)	U/mL	Real	[3.750–4468]	19
Carbohydrate antigen 19‐9 (CA19‐9)	U/mL	Real	[0.600–566.100]	34
Carbohydrate antigen 72‐4 (CA72‐4)	U/mL	Real	[0.200–158.500]	240
Carcinoembryonic antigen (CEA)	ng/mL	Real	[0.200–138.800]	22
Chlorine (CL)	mmol/L	Real	[84.600–109.400]	0
Carbon dioxide‐combining power (CO2CP)	mmol/L	Real	[16.200–34.300]	1
Creatinine (CREA)	umol/L	Real	[38.200–114]	0
Direct bilirubin (DBIL)	umol/L	Real	[0.900–12.100]	10
Eosinophil count (EO#)	10^9^/L	Real	[0–0.400]	0
Eosinophil ratio (EO%)	10^9^/L	Real	[0–7.600]	0
Gama glutamyl transferase (GGT)	u/L	Integer	[4–176]	10
Globulin (GLO)	g/L	Real	[14.100–47.600]	10
Glucose (GLU)	mmol/L	Real	[3.570–12.440]	0
Hematocrit (HCT)	L/L	Real	[0.224–0.569]	0
Human epididymis protein 4 (HE4)	pmol/L	Real	[16.710–3537.600]	20
Hemoglobin (HGB)	g/L	Real	[61.800–189]	0
Indirect bilirubin (IBIL)	umol/L	Real	[1–28.400]	10
Kalium (K)	mmol/L	Real	[3.080–5.400]	0
Lymphocyte count (LYM#)	10^9^/L	Real	[0.350–3.490]	0
Lymphocyte ratio (LYM%)	%	Real	[3.900–51.600]	0
Mean corpuscular hemoglobin (MCH)	pg	Real	[17.700–36.800]	0
Mean corpuscular volume (MCV)	fL	Real	[61–107.900]	0
Magnesium (Mg)	mmol/L	Real	[0.650–1.370]	0
Mononuclear cell count (MONO#)	10^9^/L	Real	[0.070–0.970]	0
Monocyte ratio (MONO%)	%	Real	[0.300–21.300]	0
Mean platelet volume (MPV)	fL	Real	[5.060–14.500]	2
Natrium (Na)	mmol/L	Real	[125.100–150.700]	0
Neutrophil ratio (NEU)	%	Real	[37.200–92]	91
Thrombocytocrit (PCT)	L/L	Real	[0.070–0.690]	2
Platelet distribution width (PDW)	%	Real	[8.800–22.800]	2
Phosphorus (PHOS)	mmol/L	Real	[0.570–1.750]	0
Platelet count (PLT)	10^9^/L	Integer	[74–868]	0
Red blood cell count (RBC)	10^12^/L	Real	[2.620–6.740]	0
Red blood cell distribution width (RDW)	%	Real	[10.920–22.200]	0
Total bilirubin (TBIL)	μmol/L	Real	[2.500–38.300]	10
Total protein (TP)	g/L	Real	[32.900–86.800]	10
Uric acid (UA)	μmol/L	Real	[96–632]	0
TYPE	‐	Binominal	[0, 1]	0

### 2.2 Data analysis

**Figure 1:**
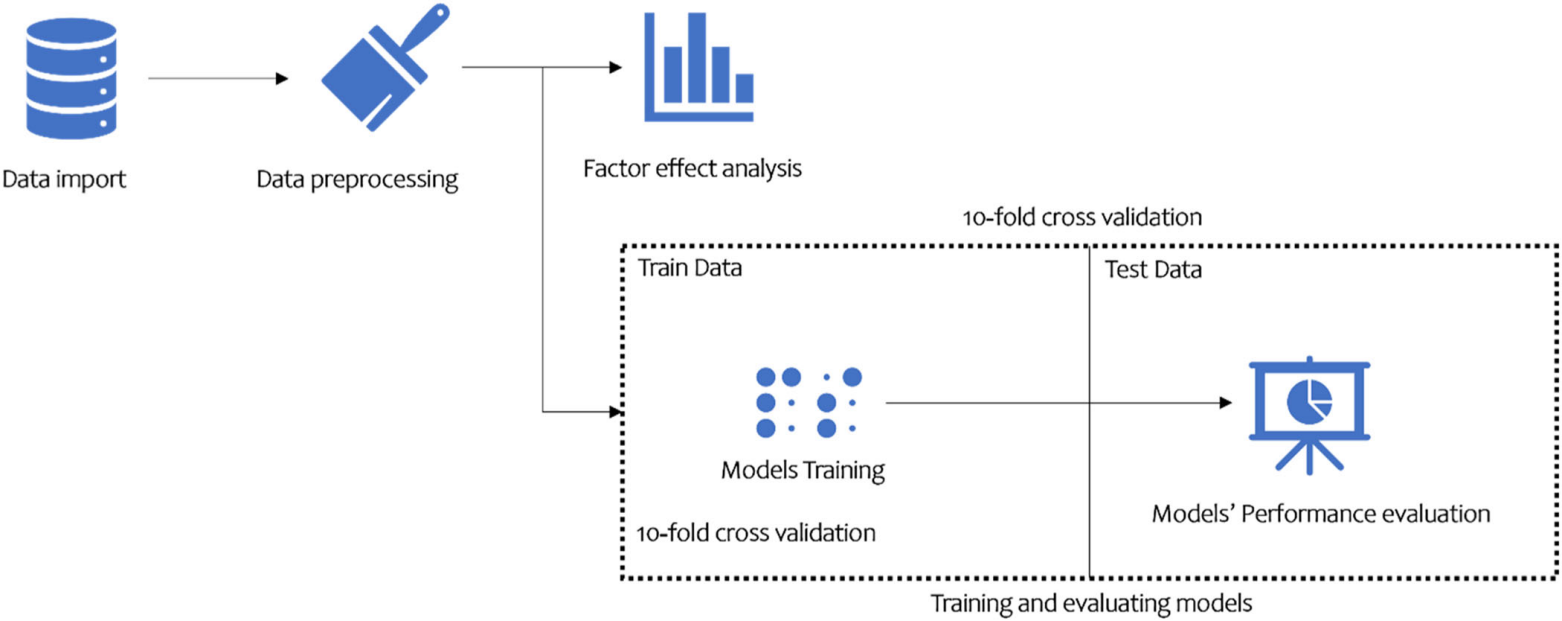
Research methodology.

**Figure 2:**
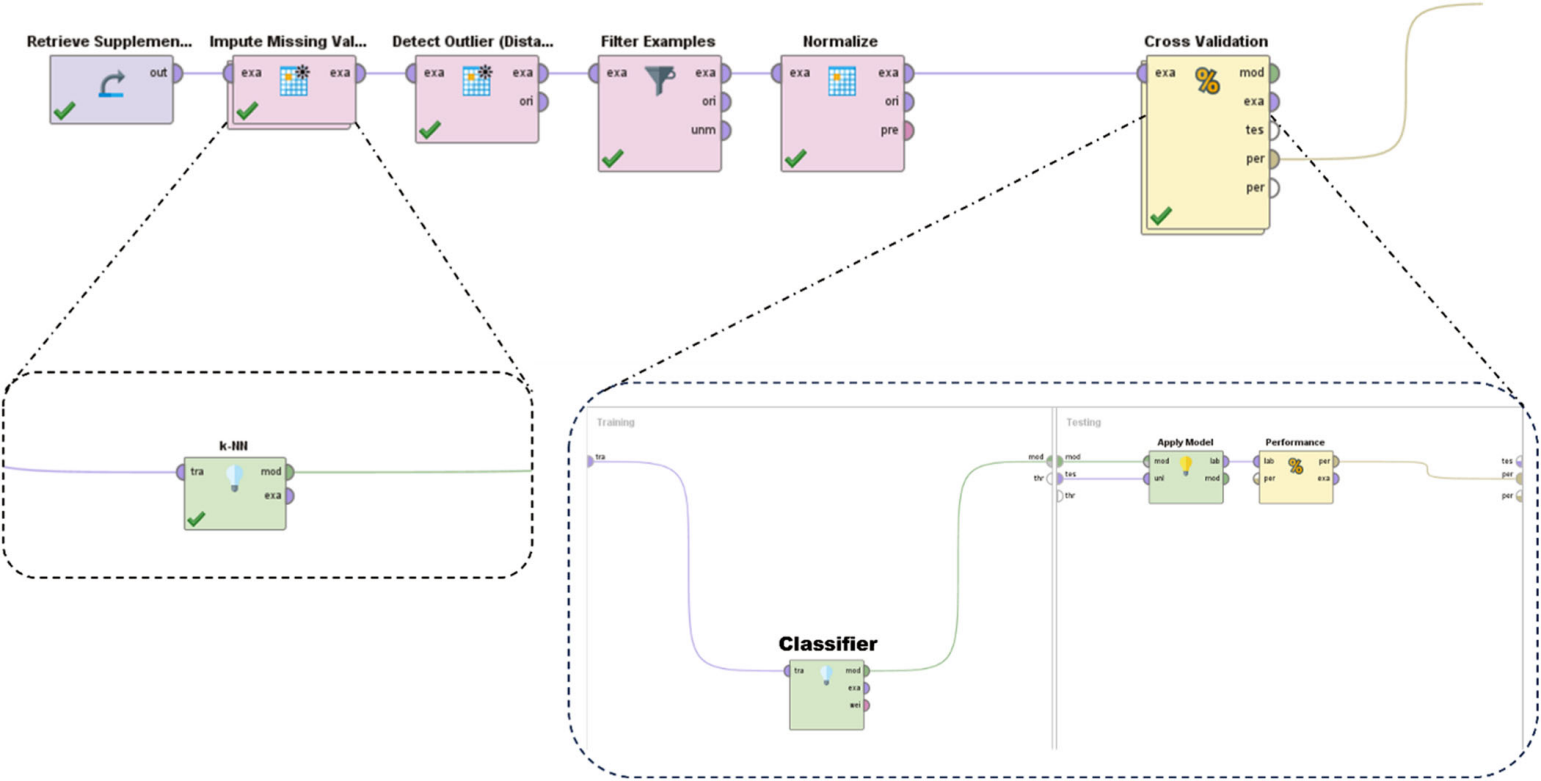
The process designed in RapidMiner software to evaluate predictive models.

The overall research steps are illustrated in Figure 1. The data analysis methodology involved the following steps:

1. Data preprocessing: The RapidMiner version 9.10 software was used to clean the data by replacing missing values, removing outliers, and normalization of the data. This step was crucial to ensure the accuracy of the subsequent analyses.
2. Factor weight determination: The weight of factors affecting ovarian cancer was determined using Information Gain (IG) and Gini Index methods. These methods helped to identify the most important factors that contribute to the development of ovarian cancer.
3. Modeling: AI models were created using classification techniques such as random forest (RF), support vector machine (SVM), and decision tree (DT). The efficiency of the models was estimated using the indicators of accuracy, sensitivity, specificity, and area under the receiver operating characteristic (ROC) curve. The models were evaluated using 10‐fold cross‐validation by specificity, sensitivity, accuracy, and ROC AUC indexes. The best model was selected based on its efficiency. The implemented blocks in RapidMiner studio are presented in Figure 2.

### 2.3 DT

DT is a machine‐learning method that makes decisions based on the graphic structure of a DT. In this method, each node of the DT represents an attribute, and the tree is created based on the relationship between the attributes. Typically, a DT is formed using a set of training data. During training, the DT algorithm identifies the optimal attribute for data splitting using metrics like entropy or Gini impurity. The objective is to maximize IG or minimize impurity after the split. By following each ray from the root to the terminal nodes, the samples move from the leaves to the root, and the final classification is determined based on the label of the leaves for each sample. The DT method has several advantages, including simplicity and high comprehensibility, the ability to check important features, the ability to use discrete and continuous input data, the ability to estimate any type of feature, and finally, the ability to check and evaluate complex conditions. DTs classify examples by traversing the tree from the root to a leaf or terminal node, where the final classification of the example is determined. The DT algorithm can be used for both classification tasks and regression tasks. Since DTs replicate human thought processes, data scientists usually find it straightforward to comprehend and interpret the outcomes. DT algorithms possess significant capabilities in data classification and evaluating the expenses, risks, and potential advantages associated with concepts.^14^, ^15^

### 2.4 RF

RF is a machine‐learning method that combines several DTs. In this method, a random forest consists of several DTs, each of which is trained independently using a random subset of features and data. The main advantage of random forest is that by combining multitree decisions, it avoids single‐tree decisions that may be incomplete, innumerable, and highly dependent on the training data. This method can be very useful and powerful for cases where the number of features is large and changeable. The RF algorithm constructs a forest by training DTs using bagging (bootstrap aggregating). Bagging is an ensemble meta‐algorithm that enhances the accuracy of ML models. The algorithm determines the final prediction by averaging the outputs from multiple trees. Increasing the number of trees improves the precision of the outcome. The ensemble of trees outputs the mode or mean of individual trees, resulting in greater accuracy and stability by leveraging multiple trees instead of relying on a single DT.^16^, ^17^

### 2.5 SVM

SVM is an ML method that has wide applications in the field of computer vision, pattern recognition, classification, and regression. The main working principle of SVM is to separate different data using a decision boundary. In the simplest case, SVM tries to create a decision boundary between the two sets of data. This decision boundary should be defined in such a way that there is the greatest possible distance between the data of each category and the boundary. We call this distance “margin.” The performance of SVM in data separation is by training a model using the training data set and finding SVMs (key points in creating the decision boundary) based on the optimization process. The distance of the closest points from both categories to the border is called the margin, and after training, SVM is able to predict new data using the decision border. One of the interesting features of SVM is that it is capable of semisupervised or nonlinear data separation using a function called the kernel function. Kernel function (such as Gaussian function or polynomial function) maps some data to a higher dimensional space so that linear separation is possible in this space. As a robust and valid algorithm, SVM performs very well in many classification and regression problems.^18^, ^19^

### 2.6 IG

IG, an entropy‐based feature evaluation technique, is extensively employed in the field of ML. Specifically, it quantifies the information contributed by feature items to a given category during feature selection.^20^, ^21^

### 2.7 Gini index

The Gini index has demonstrated its effectiveness in identifying relevant features across various applications, including ML. Widely adopted in DT algorithms such as classification and regression tree and RF, the Gini index serves as a popular technique for feature selection. Additionally, a weighted Gini index can be used as a splitting criterion to address imbalanced data.^22^, ^23^

## 3 RESULTS

### 3.1 Data set

**Figure 3:**
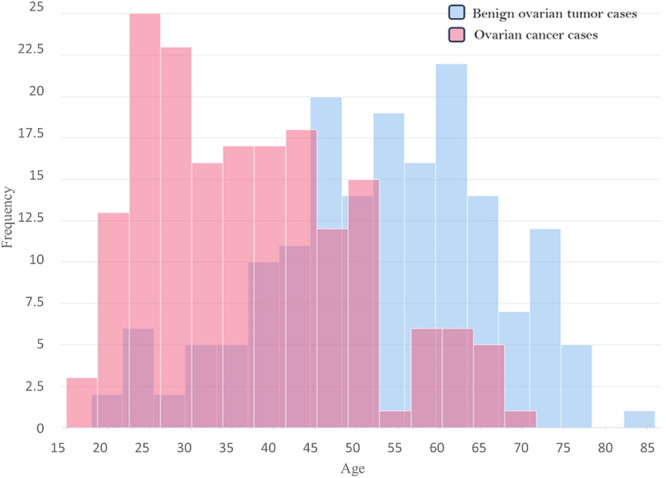
Age distribution of patients with two groups of malignant ovarian cancer and benign ovarian tumor.

This data set includes records related to 171 cases of benign ovarian tumors and 178 records related to cases of ovarian cancer. Figure 3 depicts the age distribution of both groups. The age of the samples is between 15 and 83, the average age of the samples is 45 years and their standard deviation is 15.1.

### 3.2 Analysis factors affecting the differential diagnosis of ovarian cancer

**Figure 4:**
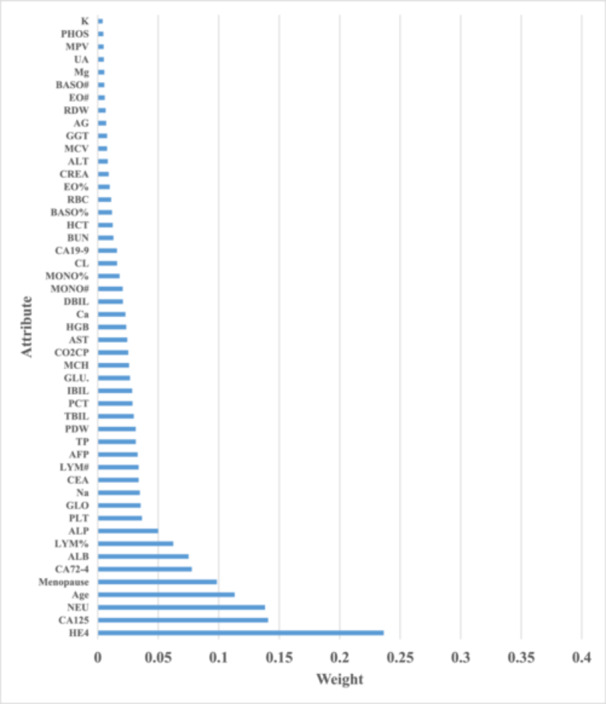
Factors affecting the diagnosis of malignant ovarian cancer obtained by the Information Gain method.

**Figure 5:**
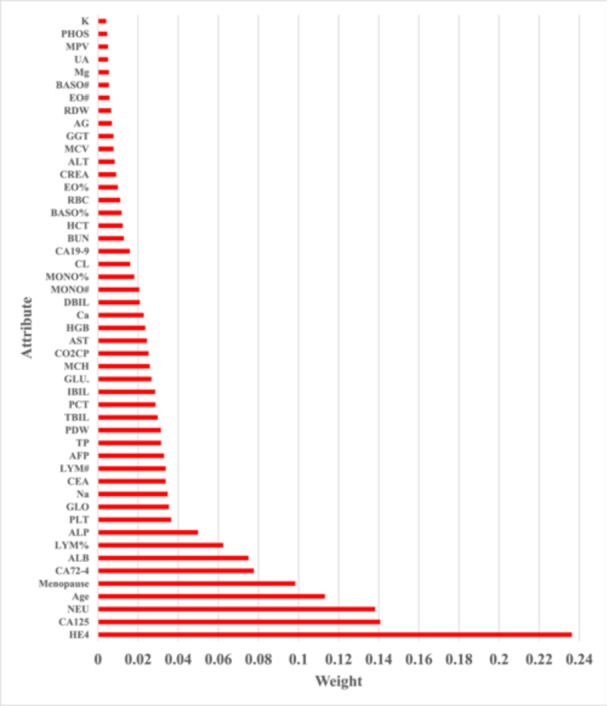
Factors affecting the diagnosis of malignant ovarian cancer obtained by the Gini Index method.

The effective factors obtained by the IG method in the diagnosis of ovarian cancer malignancy are indicated in Figure 4 and the Gini Index method in Figure 5. Three of the most important influential factors in both IG and Gini Index techniques are HE4, CA125, and NEU.

### 3.3 Evaluation of the effectiveness of predictive models for ovarian cancer

**Figure 6:**
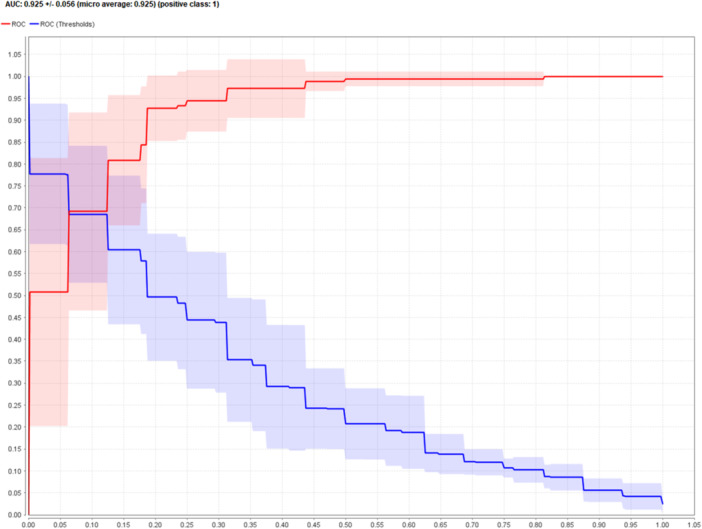
Receiver operating characteristic curve diagram of random forest model.

A comparison of the performance of these models based on accuracy, specificity, sensitivity, and area under the ROC curve is provided in Table 2. The RF model was identified as the best predictive model with an accuracy of more than 85%. The ROC diagram of the best model (RF) is presented in Figure 6.

**Table 2:** Comparing the performance of ovarian cancer prediction models.

Model	Accuracy	AUC	F measure	Sensitivity	Specificity
Decision tree	0.8291	0.799	0.8467	0.8882	0.7636
Random forest	**0.8675**	**0.925**	**0.8801**	0.9160	**0.8136**
Support vector machine	0.8525	0.910	0.8712	**0.9327**	0.7636
AutoMLP (ANN)	0.7935	0.890	0.8169	0.8706	0.7074

*Note*: The highest value of each performance index is shown in bold.

## 4 DISCUSSION

In this study, the influencing factors on the differential diagnosis of ovarian cancer were investigated, and among the 49 investigated characteristics, the most effective factors obtained by the IG method and Gini index, respectively, are HE4, CA125, NEU, and age. In addition, based on the available data, DT, SVM, RF, and ANN models were generated and compared in terms of accuracy, sensitivity, specificity, f‐measure, and AUC parameters, that the random forest model was able to provide the highest performance compared to other models.

HE4 is a protein synthesized by the majority of epithelial ovarian cancer cells, although not all cells produce it. An HE4 test can be employed to monitor epithelial ovarian cancer posttreatment, detect recurrence or disease progression, but it is not recommended for screening asymptomatic women for ovarian cancer.^24^, ^25^ High levels of HE4 are commonly found in the blood of women with epithelial ovarian cancer. Elevated HE4 concentrations can serve as an indicator for monitoring treatment effectiveness and detecting cancer recurrence or progression.^26^, ^27^, ^28^ Multiple studies have shown that the HE4 biomarker is not only valuable for diagnosing ovarian cancer but also for predicting prognosis and guiding therapy selection in patients.^24^, ^28^ Furthermore, CA125 is widely recognized as a crucial biomarker for monitoring epithelial ovarian cancer. As a screening indicator, CA125 is employed to identify patients with ovarian cancer within the population and to distinguish it from benign conditions.^29^ CA125 has been a pivotal factor in the screening, treatment, and posttreatment monitoring stages of managing ovarian cancer.^30^ HE4 and CA125 are also frequently adopted in the diagnosis of ovarian cancer.^31^, ^32^, ^33^ Although CA125 can be used to diagnose ovarian cancer, modeling using ML methods and using other factors in addition to CA125 can help create predictive models with higher accuracy (compared to pure CA125). The findings of this study align with those of similar studies.^28^, ^34^, ^35^

Similar studies have been conducted using ML methods in the field of ovarian cancer. For example, Ma et al. developed ML models to predict ovarian cancer by leveraging biomarkers, including circulating tumor cells (CTC). The best predictive model of the RF method was reported. This model achieved an approximately 80% area under the ROC curve in predicting ovarian cancer.^36^ Thus, in this study RF model with other common models were built and evaluated.

In another study conducted by Lu et al., only the SVM model was used to predict ovarian cancer and its recurrence. The results showed that the group that had a higher response rate to the chemotherapy drug exhibited recurrence in a longer time. The SVM model was able to show a sensitivity of over 90%. The limitation of this study is not using other models and not examining other parameters related to the performance of the model.^11^

Lu et al. developed ML models to predict ovarian cancer based on data from medical images of 364 patients. In this study, mathematical descriptors based on ML were used. The conclusion was that the descriptors used to predict ovarian cancer did not show a high prognostic power, but due to being noninvasive, and faster than clinical methods, they are of interest.^12^ In addition, studies such as the study of Akter et al. used methods other than biomarkers based on vaginal ultrasound screening images using ML methods have achieved favorable results.^37^ Sorayaie Azar et al. found that RF had the best performance for predicting survival in patients with ovarian cancer.^38^ Moreover, Ahamad et al. found that RF had a high performance for early‐stage detection of ovarian cancer.^39^ The results of their studies align with the findings of our study.

We removed 10 data outliers from our data set due to the possibility of biasing the model's performance. Jin et al. also removed outliers in their study, stating that their removal can significantly increase the prediction capabilities of models.^40^ Some studies have stated that removing outliers can improve the performance of classification models when mining health data.^41^, ^42^, ^43^

The current study could introduce high‐performance ML models that can be utilized for predicting ovarian cancer and overcome the limitations and disadvantages of clinical methods. This study has some limitations. First, the sample size of the data set used in the study was small, which may have limited the statistical power and generalizability of the results. Second, the data set used in the study was from a single center, which may limit the generalizability of the findings to other populations or settings.

## 5 CONCLUSION

Various AI tools have emerged as efficient tools based on available data to predict various cancers. These models can help specialists or patients in decision support systems. AI algorithms that forecast survival rates and prognoses in cancer patients provide cost‐effective support for medical decision‐making. In this study, it was concluded that different artificial intelligence tools can be used with high accuracy and sensitivity in predicting ovarian cancer. Therefore, the use of these tools can help specialists and patients with early, easier, and less expensive diagnosis of ovarian cancer. AI and ML are increasingly being used in cancer imaging, precision oncology, and cancer diagnosis and screening. AI‐based algorithms can predict treatment responses and provide robust computational tools for investigating cancer biology. The utilization of AI in cancer care encompasses various aspects such as offering clinical decision assistance for cancer diagnosis and screening, analyzing medical images, and predicting different types of cancer. AI aids in identifying the cancer origin and assessing high‐risk individuals for ovarian cancer up to 3 years before diagnosis. Additionally, AI outperforms previous tools in predicting cancer survival rates. In conclusion, AI and ML exhibit promise for diagnosing, predicting, and potentially treating various medical conditions, including cancer. AI‐based algorithms provide efficient and uncomplicated solutions to aid medical professionals in their decision‐making processes, offering a cost‐effective and practical approach to improve healthcare outcomes.

## AUTHOR CONTRIBUTIONS

Seyed Mohammad Ayyoubzadeh and Mahnaz Ahmadi analyzed and interpreted the data, Seyed Mohammad Ayyoubzadeh performed the analysis of the data, and All authors were contributors to writing the manuscript. All authors read and approved the final manuscript. Seyed Mohammad Ayyoubzadeh and Mahnaz Ahmadi had full access to all of the data in this study and took complete responsibility for the integrity and accuracy of the data analysis.

## CONFLICT OF INTEREST STATEMENT

The authors declare no conflict of interest.

## ETHICS STATEMENT

The project was found to be in accordance to the ethical principles and the national norms and standards for conducting Medical Research in Iran with Approval ID IR.TUMS.SPH.REC.1401.277 evaluated by Research Ethics Committees of School of Public Health & Allied Medical Sciences—Tehran University of Medical Sciences on February 27, 2023.

## TRANSPARENCY STATEMENT

The lead author Alireza Banaye Yazdipour, Mahnaz Ahmadi affirms that this manuscript is an honest, accurate, and transparent account of the study being reported, that no important aspects of the study have been omitted, and that any discrepancies from the study as planned (and, if relevant, registered) have been explained.

## Data Availability

All data and materials are available. Any data except the data in the manuscript can be provided upon request.
